# Caroli's Disease as a Cause of Chronic Epigastric Abdominal Pain: Two Case Reports and a Brief Review of the Literature

**DOI:** 10.7759/cureus.1701

**Published:** 2017-09-20

**Authors:** Pedro Cabral Correia, Bruno Morgado

**Affiliations:** 1 Pedro Hispano Hospital, Local Health Unit of Matosinhos; 2 Department of Biomedical Sciences and Medicine, University of Algarve

**Keywords:** caroli's disease, cholangiocarcinoma, hepatectomy, α-fetoprotein, caroli's syndrome, todani, polycystic kidney disease, bile ducts, abdominal pain, cholangitis

## Abstract

Caroli's disease is a very rare congenital malformation, currently included in cystic diseases of the biliary tract, and is characterized by ectasia and dilatation of the intrahepatic bile ducts. Two clinical entities can be distinguished, Caroli's disease in which congenital hepatic impairment is limited to cystic dilatation and Caroli's syndrome in which congenital hepatic fibrosis coexists. We present two cases of atypical presentations of Caroli's disease. Case one was a 76-year-old man who was referred to our hospital for chronic non-remitting epigastric pain prior to diagnosis. Magnetic resonance cholangiopancreatography (MRCP) was performed, which revealed findings consistent with Caroli’s disease. Laboratory investigation disclosed a raised α-fetoprotein. Left hepatectomy was performed due to suspected cholangiocarcinoma. Morphological findings were compatible with Caroli’s disease and no evidence of malignancy was found. Case two was a 47-year-old man who presented with chronic epigastric pain and generalized abdominal discomfort. MRCP revealed findings compatible with Caroli’s disease. The patient was discharged with ursodeoxycholic acid treatment and was later admitted twice due to inaugural episodes of cholangitis that were medically managed. Bisegmentectomies II and III were performed for suspected neoplasia after a gradual rise in α-fetoprotein and CA19-9 values were noted during follow-up. The surgical specimen confirmed Caroli’s disease and there was no evidence of malignancy. Postoperative periods for both patients were favorable, and they remain asymptomatic and well to date.

## Introduction and background

Caroli's disease is a rare congenital malformation of the intrahepatic bile ducts characterized by duct ectasia and dilation, which may involve the biliary tract in a focal or multifocal manner [1]. It is currently included in group V of the Todani classification of biliary tract cystic diseases and was first described by the French gastroenterologists, Jacques Caroli et al., in 1958 [2-3].

Caroli’s disease is less common than Caroli’s syndrome, and both are extremely rare with an approximate prevalence of less than one in 1,000,000 inhabitants [4]. In Caroli's disease, congenital hepatic impairment is limited to the development of cysts and usually presents with abdominal pain in the right hypochondrium, obstructive jaundice, and cholangitis. In Caroli’s syndrome, cystic disease coexists with congenital or primary hepatic fibrosis. In this case, the clinical presentation is usually due to hepatic insufficiency and portal hypertension, presenting with findings such as splenomegaly, ascites, peripheral edema, coagulation disorders, and esophageal varices [5-6]. In both Caroli’s disease and syndrome, several conditions may occur together, such as choledochal cysts, autosomal recessive polycystic kidney disease (ARPKD), or even autosomal dominant polycystic kidney disease (ADPKD), renal medullary spongiosis, and medullary cystic disease [6-7]. Both conditions less commonly affect men, with a male to female ratio of 1:1.8. Although these conditions are generally diagnosed within the first two decades of life, they may remain asymptomatic for the entire life of the affected individual [7-8].

We describe two cases of Caroli’s disease presenting with chronic, low-grade, abdominal pain. We believe the present reports are of medical significance since they relate to patients with atypical presentations and diagnosed at an unusually late age. It serves also as a reminder that although rarely, Caroli’s disease may present later in life, have atypical presentations and be masked by non-specific clinical findings. These reports hope to add to the existing knowledge base of this very rare congenital disease.

## Review

Case one

**Figure 1:**
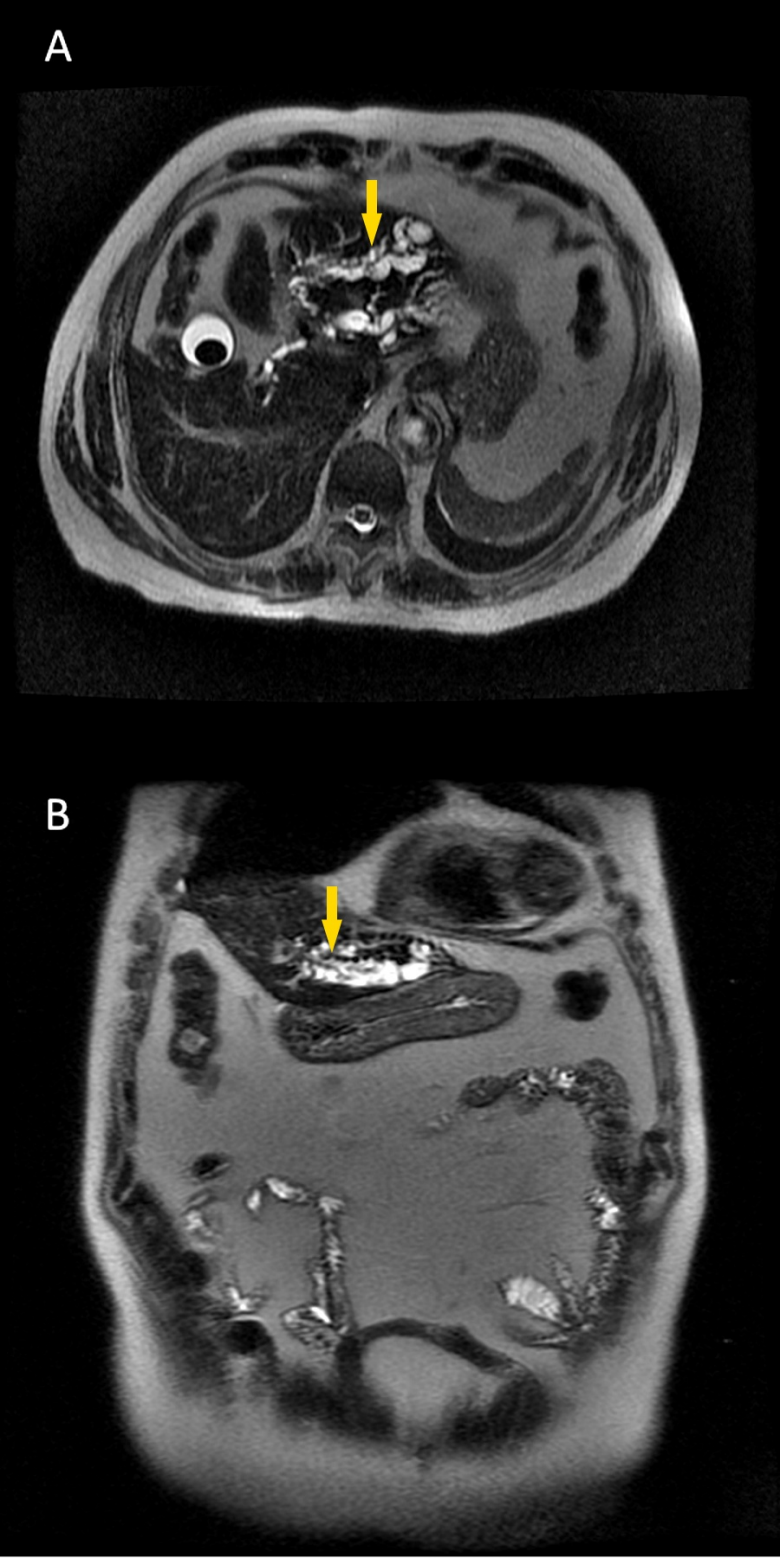
Magnetic resonance cholangiopancreatography showing (A) saccular dilatation of the left biliary tree (arrow) and (B) biliary dilatation in the left hepatic lobe (arrow).

A 76-year-old man consulted his primary care physician due to low-grade, chronic, epigastric pain with additional non-specific complaints. The pain was pressing in character, non-remitting and had been present for approximately eight months. The patient denied nausea, vomiting, weight loss, changes in bowel habits or fecal characteristics, fever, or urinary symptoms. He also denied alcohol or tobacco consumption. His history was positive for dyslipidemia, hypertension, obesity, orthopedic surgery in the right knee at age 39 due to trauma, and radical prostatectomy at age 69 following the diagnosis of a prostatic carcinoma. Abdominal ultrasonography revealed vesicular lithiasis, as well as a heterogeneous nodular lesion and atrophy of the left lobe of the liver. The patient was therefore referred for surgical consultation to assess the need for cholecystectomy and to further investigate the liver findings. A physical examination was remarkable for an overweight patient (weight 70 kg, 1.65 m high), blood pressure of 146/92 mmHg, and pulse of 68/min. An abdominal examination revealed a non-painful abdomen with positive bowel sounds, with no discernible organomegaly. The rest of the examination was unremarkable, as the patient presented systemically well with all observations within normal range. A magnetic resonance cholangiopancreatography (MRCP) was performed, which revealed dilatation of the left intrahepatic bile ducts associated with parenchymal atrophy (Figures 1A, 1B).

Three nodular images without phase enhancement in the dynamic study were also identified, the first with 20 mm in the left branch of the main bile duct at the level of the bifurcation, the second with 12 mm in the duct of segment II, and a third downstream of the previous one with 5 mm. These lesions were described as being compatible with a possible cholangiocarcinoma. The right bile duct presented a small ectasia with 4.3 mm without parenchymal or upstream bile duct changes. The extrahepatic bile ducts were unremarkable for changes in morphology or duct caliber. A cystic lesion of the head of the pancreas and several parapyelic and cortical renal cysts were also observed.

Laboratory data of peripheral blood on admission are provided in Table 1.

**Table 1:** Laboratory data of peripheral blood on admission.

Laboratory test	Result
Hb (g/dl)	16.2
WBC x 10^9^/L	8.5
Plt (x10^4^/mm^3^)	187
PT (INR)	1
aPTT (s)	28.4
T-Bil (mg/dl)	2
D-Bil (mg/dl)	0.6
Creatinine (mg/dl)	1
Alkaline phosphatase (U/l)	61
AST (U/l)	30
ALT (U/l)	28
Amylase (U/l)	83
CRP (mg/dl)	0.14
α-fetoprotein (ng/ml)	14.89
CEA (ng/ml)	0.72
CA19-9 (IU/ml)	5.86

**Figure 2:**
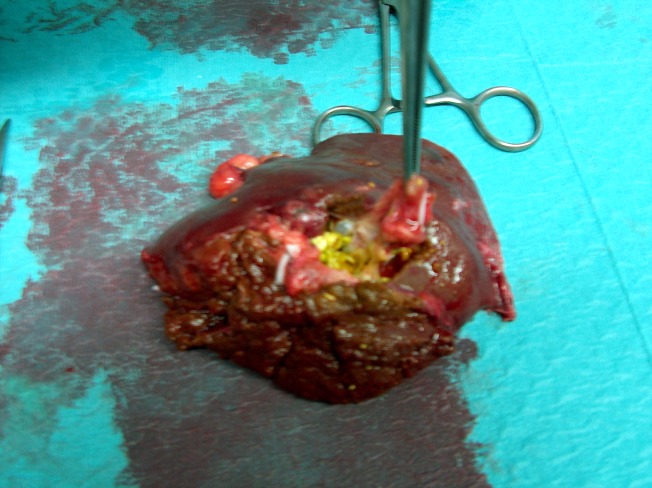
Surgical specimen showing cysts and gallstones.

Investigations disclosed an elevated α-fetoprotein level and due to the image and laboratory findings, a left hepatectomy was deemed appropriate. Histological examination of the excised portion (Figure 2) revealed segments of the liver with parenchymal atrophy, multifocal cystic dilatation of the intrahepatic bile ducts, periductal fibrosis, and an inflammatory infiltrate.

Discrete cholestasis, foci of micro- and macro-vesicular steatosis, scattered lymphocytes, and intrahepatic lithiasis were also observed. The lymph nodes showed no histological changes nor did the surgical specimen present with histopathological findings of malignancy. The morphological findings described were compatible with Caroli’s disease with unilobar involvement and secondary periductal liver fibrosis.

The postoperative period was favorable and without complications, and the patient was discharged seven days later. Post-operatively, due to age, the exclusion of neoplastic suspicion and a favorable clinical outcome, follow-up benefits, and a proposal of discharge from the outpatient clinic was discussed, which the patient agreed to, remaining asymptomatic, under surveillance in the primary care setting.

Case two

**Figure 3:**
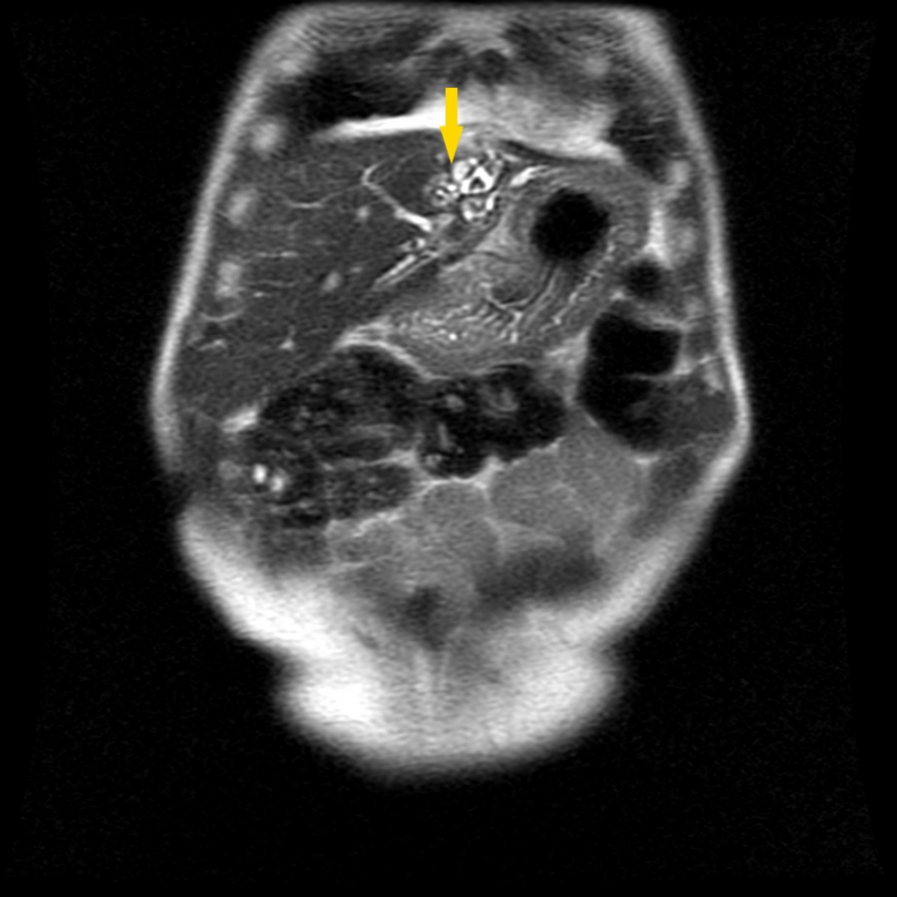
Magnetic resonance cholangiopancreatography showing intrahepatic biliary ectasia (arrow).

A 47-year-old man presented to his primary care physician with low-grade, chronic epigastric abdominal pain, which had lasted for six months, and was referenced to our department for further evaluation. The pain was described as pressing in character, with no irradiation or associated factors. His history was positive for oligophrenia, congenital malformation of the upper limbs, bilateral cataracts, chronic hepatitis B, type I diabetes mellitus, and cholecystectomy at age 38. The patient was mildly tender, locally, over the epigastric region of the abdomen but rebound tenderness was absent. Abdominal ultrasonography exposed an apparent atrophy of the left lobe of the liver. MRCP revealed atrophy of the left lobe, dilation of the intrahepatic biliary tract and intrahepatic lithiasis, as well as a renal cyst on the left (Figure 3).

**Figure 4:**
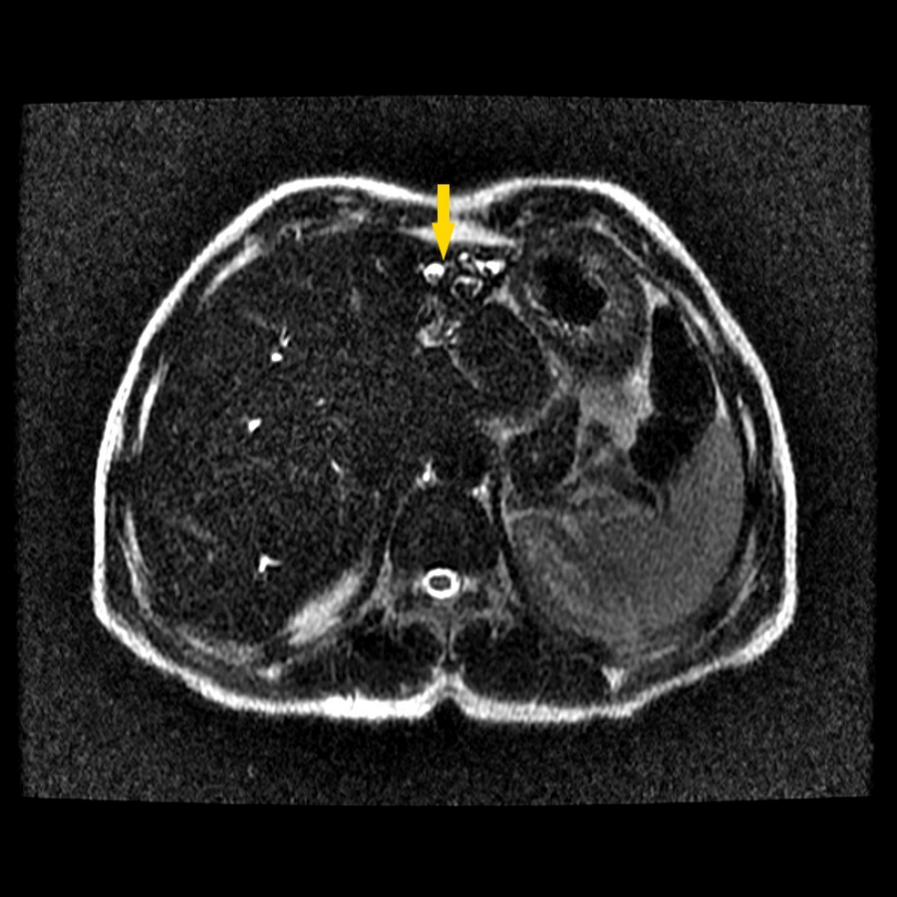
Magnetic resonance cholangiopancreatography showing lithiasis of the main bile duct (arrow).

Based on these findings, a diagnosis of Caroli's disease was made. The patient was prescribed ursodeoxycholic acid and discharged with follow-up appointments at the outpatient clinic with analytical reassessment and an annual MRCP. Seven months later, he presented to the emergency department with fever and severe abdominal pain. An MRCP was performed, which in addition to the previous findings, revealed lithiasis of the main biliary tract (Figure 4).

A presumptive diagnosis of cholangitis was made, and he was admitted for seven days for medical management with antibiotic therapy. An upper endoscopy was performed, which revealed no abnormal findings, and an abdominal CT revealed pneumobilia in the left intrahepatic biliary tracts and a small cortical cyst in the left kidney. Five years later, he had a second episode of cholangitis, was admitted to our department, and, once again, was medically treated for seven days and discharged for follow-up. In the time between the acute episodes, the patient complained only of low-grade, chronic epigastric abdominal pain, which was refractory to analgesic or ursodeoxycholic acid treatment.

Laboratory data of peripheral blood during follow-up after the last cholagitis episode are provided in Table 2.

**Table 2:** Laboratory data of peripheral blood during follow-up.

Laboratory test	Result at 6 months	Result at 12 months	Result at 16 months
Hb (g/dl)	13.6	14.5	13.5
WBC (x10^9^/L)	5.6	4.3	4.6
Plt (x​10^4^/mm^3^)	148	153	159
PT (INR)	1.0	1.1	1.1
aPTT (s)	24.2	23.1	25.2
Glycemia (mg/dl)	374	381	393
LDH (U/l)	162	151	163
Creatinine (mg/dl)	0.8	0.7	0.7
Alkaline phosphatase (U/l)	132	131	135
G-GT (U/l)	98	101	99
TGO (U/l)	15	17	16
T3 (ng/ml)	0.87	0.9	0.91
TSH (IU/ml)	1.23	1.38	1.43
α-fetoprotein (ng/ml)	5.79	7.21	13.11
CEA (ng/ml)	1.2	1.4	1.5
CA19-9 (IU/ml)	5.26	6.65	12.43

The gradual increase of α-fetoprotein and CA19-9 noted during follow-up, together with a history of hepatitis and Caroli's disease, supported the suspicion of neoplasia, and a surgical proposal was made. The patient was hospitalized and operated, having undergone bisegmentectomies II and III. During the postoperative period, a bile collection was found adjacent to the sectioned area that required percutaneous drainage with a pigtail catheter and two instances of endoscopic retrograde cholangio-pancreatography were used to extract gallstones and remove biliary sludge that obstructed the common bile duct. An examination of the surgical specimen demonstrated a rough capsular surface with an ill-defined area of 5x5x4 cm, with dilated ducts, wall fibrosis, multifocal hyperplasia of the peribiliary glands, and a lymphoplasmacytic infiltrate. The lumen was occupied by friable brownish calculi, therefore, confirming intrahepatic lithiasis in Caroli's disease, and there was no evidence of malignancy.

While maintaining biliary drainage, he was discharged on the 35th day to the outpatient clinic, where the pigtail catheter was eventually removed. The patient remains asymptomatic but continues to frequent the outpatient clinic for imaging follow-up of a residual biloma. An MRCP performed four years later demonstrates that although there are no parenchymal focal lesions, there is a slight dilatation of the intrahepatic biliary tract and some endoluminal focal filling defects, as well as a simple cortical cyst in the left kidney of 18 mm.

Discussion

The cause of Caroli’s disease is still unknown, but a genetic profile appears to exist and seems autosomal recessive in Caroli syndrome and sporadic in Caroli's disease [9]. Studied mutations include genes that participate in the development of the kidneys and biliary tree, the most important of which is the polycystic kidney and hepatic disease 1. This gene is thought to participate in the regulation of cell proliferation and adhesion, highly expressed in the kidneys and expressed in lower levels in the pancreas, liver, and lungs [9-12]. Mutations in the PKD1 and PKD2 genes are less frequent and appear to be the basis of several renal and hepatic abnormalities [10].

Caroli's disease, when symptomatic, usually manifests with recurrent episodes of upper right quadrant abdominal pain, which may be accompanied by pruritus and jaundice. Recurrent bacterial cholangitis is the most common form of presentation, which may present as abdominal pain, fever, rigors, and malaise [5,13-14]. The clinical presentation in our cases was atypical, presenting mainly with chronic pain and nonspecific complaints. In case one, the patient reported low-grade, chronic, epigastric abdominal pain and nonspecific symptoms, which prompted image studies that incidentally detected cystic lesions compatible with Caroli’s disease. In case two, the patient presented similarly but more typical recurrent episodes of cholangitis followed, which led to hospitalization and additional investigations. Both patients in our cases became symptomatic at an unusual age. Typically, inaugural symptomatic episodes usually start between adolescence and early adulthood but can appear at any time. A small number of patients may remain asymptomatic through life and may be diagnosed accidentally by imaging tests, even at older ages [9,13,15].

Among the most common ancillary tests used, ultrasound is the best initial examination since it is non-invasive, fast, and cheap, despite being operator-sensitive and having low specificity. Ultrasound findings are characterized by the irregular dilatation of the intrahepatic bile ducts, sometimes accompanied by extrahepatic ductal dilation due to cholelithiasis. Ultrasound also allows for renal evaluation in search of evidence of polycystic kidney disease [7,11,16-17]. An MRCP with high sensitivity, specificity, and low associated invasiveness is currently the examination of choice. Though more expensive, it is universally considered the most consistent method for assessing disease extent and severity [5,8,18].

No guidelines exist for Caroli’s disease and given the scarcity of case reports, there are no randomized trials on different treatment modalities. Proposed therapy should, however, be tailored to individual patients and be contingent on clinical presentation, site, and extent of biliary abnormalities [19]. Medical therapy should be implemented with ursodeoxycholic acid, which acts to decrease hepatic synthesis, secretion, and intestinal absorption of cholesterol, consequently increasing bile fluidity [5,20-21]. Aggressive surgical strategies seem to provide the best results when complications, cancer risk, or disease extent are enough to justify a surgical procedure [22-24]. Surgical indication in these two cases of Caroli's disease was the suspicion of malignant neoplasia, expressed in the MRCP findings in case one and in the gradual increase of tumor markers (α-fetoprotein and CA19-9) associated with a history of hepatitis in case two. In case one, a left hepatectomy was performed and the evolution was frankly favorable with an apparent resolution of the underlying symptomatology and disease. In case two, a left lateral bisegmentectomy was performed and although the patient is asymptomatic, imaging findings suggestive of vestigial biloma remain in the hepatic remnant. Because the risks of recurrence and neoplastic conversion are not permanently removed, annual follow-up is maintained at the hepato-pancreato-biliary outpatient clinic. Both patients maintain complete symptomatic resolution with surgical treatment until the present day.

Patients with Caroli’s disease usually require close follow-up and regular abdominal ultrasound, blood count measurement, and markers of inflammation, liver function, and injury are usually advised [5,13,21-22,25]. In the event of exacerbations or in the emergence of complications, hospitalization should be weighed. Cholangitis, sepsis, cholelithiasis requiring interventional therapy, and complications from portal hypertension are indications for inpatient treatment. A liver biopsy should also be performed in the inpatient setting when disease suspicion outweighs the morbidity associated with this technique [4,8,13,19].

There are no unanimously accepted protocols for cholangiocarcinoma screening. It is, however, consensual that ancillary tests, such as abdominal ultrasound, tumor markers (CA19-9 and CEA), or MRCP should be regularly performed given the high risk in this patient population [24]. Genetic counseling and familial investigation are also advised during follow-up in patients with Caroli’s disease [6].

## Conclusions

In summary, Caroli’s disease is a rare congenital malformation of the intrahepatic bile ducts and may be associated with conditions affecting other organ systems, such as polycystic kidney disease. Despite the very low prevalence, Caroli’s disease should be present in the differential diagnosis of epigastric abdominal pain and recurrent cholangitis without risk factors or relevant history. After clinical suspicion, the diagnosis is always imagiologic, with ultrasound and MRCP being the tests of choice. Caroli’s disease may present with indolent abdominal pain instead of the more typical presentation of recurrent cholangitis. Medical therapy should be performed with ursodeoxycholic acid, but surgical intervention can be curative. Nevertheless, the management strategy should be adapted to individual cases and strongly depends on the location and extent of cystic dilations, history of the disease, complications, and comorbidities. Complications can be frequent and severe, and mortality from cholangiocarcinoma deserves early intervention and close follow-up.
